# Nuclei of Non-Muscle Cells Bind Centrosome Proteins upon Fusion with Differentiating Myoblasts

**DOI:** 10.1371/journal.pone.0008303

**Published:** 2009-12-14

**Authors:** Xavier Fant, Vlastimil Srsen, Aude Espigat-Georger, Andreas Merdes

**Affiliations:** 1 Wellcome Trust Centre for Cell Biology, University of Edinburgh, Edinburgh, United Kingdom; 2 Unité Mixte de Recherche 2587, Centre National de la Recherche Scientifique-Pierre Fabre, Toulouse, France; Katholieke Universiteit Leuven, Belgium

## Abstract

**Background:**

In differentiating myoblasts, the microtubule network is reorganized from a centrosome-bound, radial array into parallel fibres, aligned along the long axis of the cell. Concomitantly, proteins of the centrosome relocalize from the pericentriolar material to the outer surface of the nucleus. The mechanisms that govern this relocalization are largely unknown.

**Methodology:**

In this study, we perform experiments in vitro and in cell culture indicating that microtubule nucleation at the centrosome is reduced during myoblast differentiation, while nucleation at the nuclear surface increases. We show in heterologous cell fusion experiments, between cultures of differentiating mouse myoblasts and human cells of non-muscular origin, that nuclei from non-muscle cells recruit centrosome proteins once fused with the differentiating myoblasts. This recruitment still occurs in the presence of cycloheximide and thus appears to be independent of new protein biosynthesis.

**Conclusions:**

Altogether, our data suggest that nuclei of undifferentiated cells have the dormant potential to bind centrosome proteins, and that this potential becomes activated during myoblast differentiation.

## Introduction

Muscle fibres are syncytia formed by fusion of differentiating myoblasts. During differentiation, the cytoskeleton of myoblasts is profoundly remodelled. Skeletal actin and myosin are organized into contractile sarcomeres. Several groups have postulated that this process depends on an initial reorganization of the microtubule network [1]–[4]. Microtubules, emanating in a radial pattern from the centrosome, are realigned into an array of fibres running parallel to the long axis of the cell [5]. Concomitantly, a large percentage of centrosome proteins are relocated from the pericentriolar material to the surface of the nucleus [6]–[8] where they form a dense, fibrillar matrix surrounding the outer nuclear membrane [8]. The residual centrosome proteins appear to remain bound to the pericentriolar material, and part of these proteins are also seen finely dispersed in the cytoplasm [7]. Clusters of “centrosomal elements” are sometimes found around the nuclei in fused myotubes, and these “centrosomal elements” are believed to retain centrioles [7]. During differentiation, relocation of proteins from the pericentriolar material to the nucleus starts at an early stage, before fusion of myoblasts into myotubes [8]. It is conceivable that the relocalization of centrosome proteins is a prerequisite for the reorganization of the microtubule network. So far, the molecular mechanisms leading to the relocalization of centrosome proteins are not understood. In this study, we investigate how cytoplasmic factors of undifferentiated and differentiated myoblasts affect the centrosome, using in-vitro-assays and heterologous cell fusion.

## Results

### The Nuclear Surface Becomes the Predominant Site of Microtubule Nucleation in Differentiating Myoblasts

**Figure 1:**
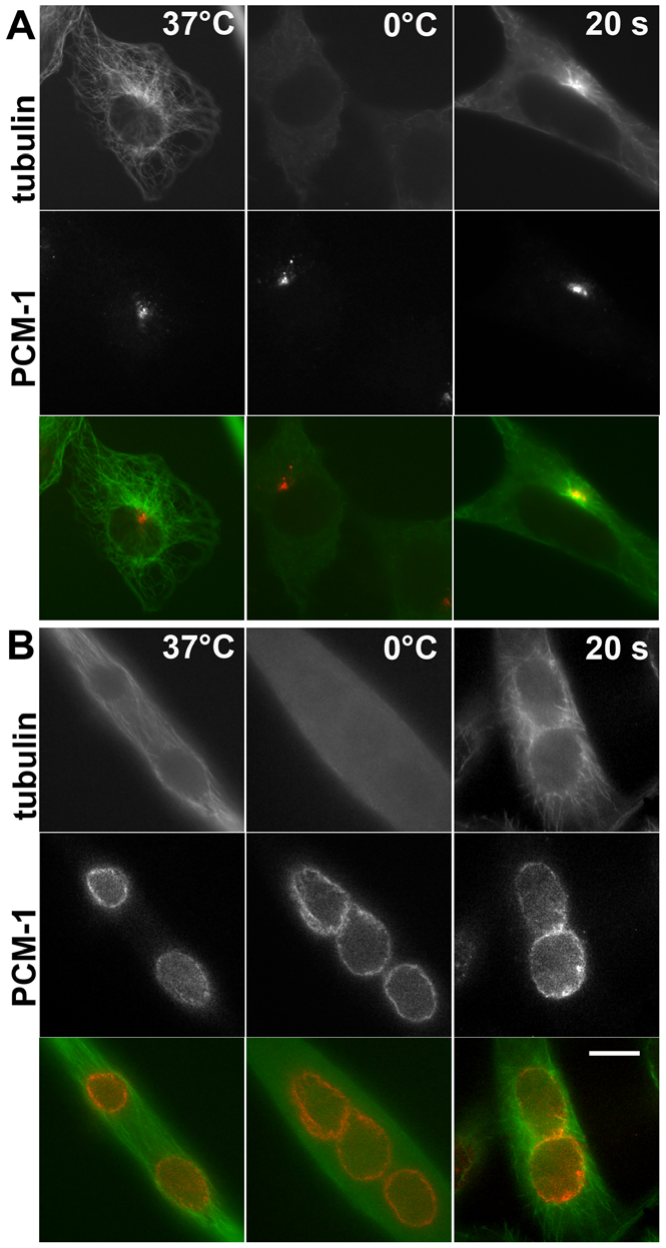
In myotubes, microtubules are nucleated from the nuclear surface. (A) Immunofluorescence of undifferentiated *H-2K* ^b^-tsA58 mouse myoblasts, and (B) differentiated, fused *H-2K* ^b^-tsA58 myotubes, stained for tubulin (green), and for the centrosome protein PCM-1 (red). Left: cells at 37°C, displaying an intact microtubule network. Middle: depolymerization of microtubules after treatment on ice for three hours. Right: microtubule re-growth, 20 seconds after shifting cells to 37°C. A small centrosomal microtubule aster is seen in the undifferentiated cell (A), whereas sun-like rays of microtubules emanating from the nuclear surface are seen in myotubes (B). Bar in B, 10 µm. Identical magnifications in A and B.

To investigate whether centrosomes in myoblasts are capable of nucleating microtubules after differentiation, we used cultured mouse *H-2K* ^b^-tsA58 myoblasts, carrying a thermolabile T-antigen, which permits differentiation upon a temperature shift from 33 to 37°C [9]–[10]. These cells were induced to differentiate for three days. As shown previously, a variety of centrosome proteins relocalizes to the nuclear surface during this differentiation process, even prior to fusion into myotubes (Fig. 1A, B) [8]. We investigated the sites of microtubule re-growth following cold-induced depolymerization (0°C for 3 hours).

In myoblasts (Fig. 1A), the microtubule network of untreated, undifferentiated myoblasts is organized by the centrosome. Cold treatment led to the disappearance of most microtubules, except for a small number of resistant, stable microtubules. Upon shifting the cells to 37°C for several seconds, new microtubule asters were nucleated by the centrosome (Fig. 1A). In comparison, differentiated *H-2K* ^b^-tsA58 cells that had fused into myotubes showed longitudinally oriented microtubules prior to depolymerization. Likewise, these microtubules disappeared after cold treatment. After re-warming, the highest density of re-growing microtubules was seen around the surface of each nucleus (Fig. 1B), consistent with a previous report [6]. The pattern of re-growing microtubules was reminiscent of sun-like arrays (Fig. 1B).

Since centrosome proteins accumulate at the nuclear surface in these differentiating cells [6]–[8], we investigated whether nuclei represented the sole microtubule organizing centres and whether the residual “centrosomal elements” (i.e. centriole-containing clusters as described in [7]) had lost their original potential of microtubule nucleation. This idea was tested in experiments using purified centrosomes and cell extracts. Since microtubule-nucleating factors can be stripped from the centrosome by treatment with high salt, we tested whether cytoplasmic extracts from undifferentiated or differentiated *H-2K* ^b^-tsA58 and C2C12 cells, 120 hours post induction of differentiation, reconstituted the microtubule-nucleating potential.

**Figure 2:**
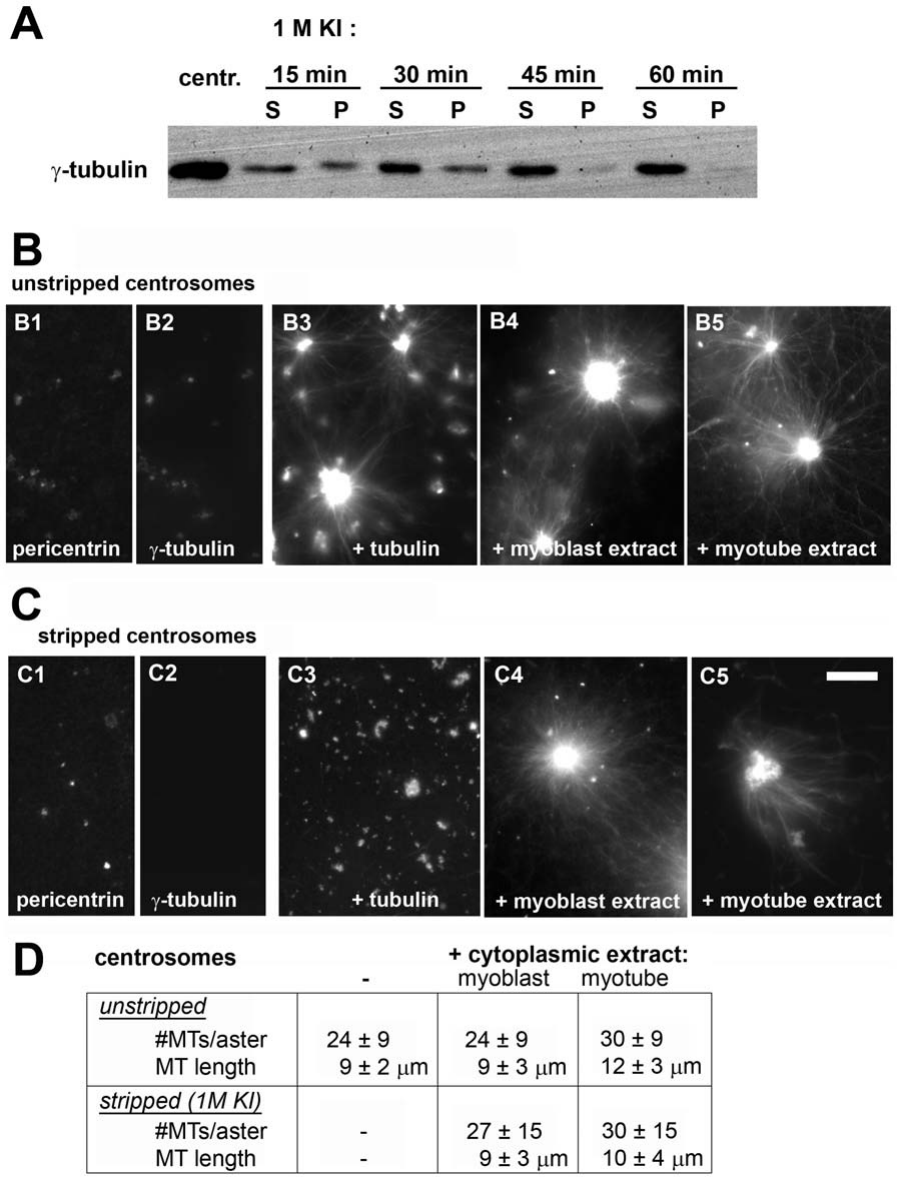
Nucleation of microtubules from salt-stripped centrosomes is restored by cytoplasmic extract of myotubes. (A) Isolated centrosomes from Jurkat cells, treated with 1M potassium iodide (KI) to strip the pericentriolar material. An immunoblot is shown, probed for gamma-tubulin in unstripped centrosomes (centr.), as well as in centrosomes after KI-treatment for 15, 30, 45, and 60 minutes. Treated centrosomes were fractionated by centrifugation, to separate the extracted pericentriolar material (supernatants, S), and the insoluble centrioles (pellets, P). (B) Purified centrosomes, centrifuged onto glass coverslips and stained for immunofluorescence of pericentrin (B1) and gamma-tubulin (B2). Centrosomes were incubated with purified tubulin at 5 mg/ml for 10 minutes, either directly (B3), or following incubation with cytoplasmic extract from undifferentiated myoblasts (B4) or from myotubes (B5). (C) Equivalent experiments as in (B), using centrosomes that had been stripped with KI for one hour. (D) The number of microtubules/aster in a focal plane, as well as the average microtubule length were quantified in each case (n≥34). Bar in (C), 10 µm.

Treatment with 1M potassium iodide for one hour removed nearly all of gamma-tubulin from purified centrosomes (Fig. 2A), whereas pericentrin remained largely bound (Fig. 2B, C). This is consistent with a proteomic analysis of the centrosome [11], demonstrating that various centrosome proteins are extracted differentially by high salt-treatment. The stripped centrosomes were unable to nucleate microtubules in a solution of purified tubulin, whereas unstripped centrosomes induced the formation of microtubule asters (Fig. 2B, C), underlining the importance of gamma-tubulin for centrosomal microtubule nucleation. However, incubation of stripped centrosomes with cytoplasmic extracts from either undifferentiated myoblasts or from myotubes restored microtubule nucleation and the formation of microtubule asters (Fig. 2C). The number of microtubules per aster, as well as the length of microtubules were similar under the different conditions (Fig. 2D): purified centrosomes tended to aggregate into clusters, which had diameters of multiple µm. These were nucleating 24 to 30 microtubules/2 µm cluster in a focal plane, with an average microtubule length of 9 to 12 µm.

**Figure 3:**
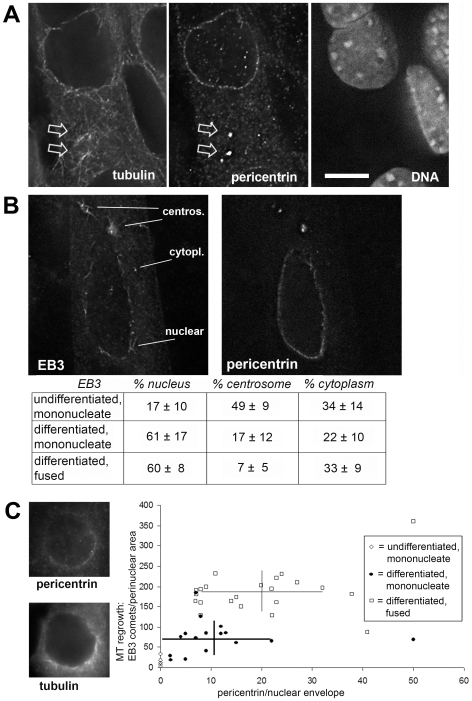
Microtubule nucleation from the centrosome is reduced in the differentiation process. (A) Deconvolved optical sections of differentiated muscle cells after re-growth of microtubules for 20 seconds, stained for immunofluorescence of (left) microtubules, (middle) the centrosome protein pericentrin, and (right) stained with the DNA marker DAPI. In a myotube early after fusion, pericentrin is visible both around the nuclear surface and on the centrosomes (arrows). Microtubule re-growth is seen from these centrosomes, in addition to perinuclear and cytoplasmic sites. (B) Myotube, after cold-induced depolymerization of microtubules and re-growth for 10 seconds. Immunofluorescence of the plus-end-binding protein EB3 and of pericentrin are shown. Cytoplasmic, nuclear, and centrosomal sites of emerging EB3 comets are indicated on the left. The table indicates the percentage of EB3 comets growing from cytoplasmic, nuclear, and centrosomal locations in undifferentiated cells (n = 6), as well as in differentiating cells before fusion (mononucleate, n = 15), and after fusion (n = 12). The total number of EB3 comets per cell ranged from 39 (undifferentiated) to 1151 (differentiated, fused). (C) Left: nucleus of a myoblast at an early stage of differentiation, stained for pericentrin (top) and microtubules (bottom), recovering for 10 seconds from cold-induced depolymerization. The number of microtubules grown from the nuclear surface was quantified by counting EB3 comets as in (B). The results for each nucleus were plotted against the intensity of perinuclear pericentrin (arbitrary values, after subtraction of background) in undifferentiated cells (n = 6), and in mononucleate (n = 15) and fused differentiated cells (n = 22). Horizontal and vertical bars indicate standard deviations of pericentrin intensity and of the number of EB3 comets, respectively, in differentiated mononucleate and fused cells. Bar in (A), 10 µm; identical magnification in (B), (C).

These experiments suggested that the cytoplasm of myotubes retained all factors necessary for microtubule nucleation. We therefore examined directly whether centrosomes maintain their potential to nucleate microtubules in living myotubes, using deconvolution microscopy. We reported previously that at early stages of differentiation, the centrosome proteins pericentrin, PCM-1 and cdk5rap2 repartition between the pericentriolar material and the outer nuclear surface [8]. In such cells, small asters of re-growing microtubules were seen at pericentrin foci, reminiscent of centrosomes (or “centrosomal elements” as described by [7]), in addition to microtubules emanating from the perinuclear surface, and free cytoplasmic microtubules (Fig. 3A). To determine the relative percentage of microtubules nucleated from the centrosome, from the nuclear surface, or from the cytoplasm, we counted growing microtubule plus-ends identified by EB3 labelling [12], after recovery from cold-induced depolymerization (Fig. 3B). We found that in undifferentiated myoblasts, there was predominant nucleation of microtubules from the centrosome (49%), whereas in differentiating cells, nucleation from the centrosome was gradually reduced from 17% to 7% after fusion into myotubes. At the same time, the nuclear surface became the dominant site of microtubule nucleation (60%).

To gain further insights, we tested whether the nucleation capacity of the nuclear surface correlated with the amount of accumulated centrosome protein (Fig. 3C). Immunofluorescence microscopy of myoblasts at early stages of differentiation showed that they contained nuclei that had accumulated pericentrin only on part of their surface, indicating that re-growing microtubules emanated mainly from areas with the highest amounts of pericentrin. Subsequently, we quantified the amount of EB3 comets emanating from the nucleus and plotted these against the intensity of pericentrin immunofluorescence at the respective nuclear surface (Fig. 3C, right). We found that perinuclear staining of pericentrin increased during the differentiation process, correlating with an increase of the number of EB3 comets within 2 µm of the nuclear envelope, from 15±11 (SD) in undifferentiated cells to 72±40 in unfused, differentiated cells. After fusion into myotubes, we detected a further increase to 187±72 comets of EB3 per nuclear surface. Although the data varied from cell to cell, overall they suggest that the number of microtubules grown off the nucleus is proportional to the amount of centrosomal material.

### Nuclei of Non-Muscle Cells Acquire Centrosome Proteins upon Experimentally Induced Cell Fusion with Myotubes

Myoblasts lose their centrosome-dependent radial microtubule organization during the differentiation process. Although it was reported that the centriole marker centrin remained present at “centrosomal elements” in mouse myotubes fused in culture [7], centrioles were described to disappear almost completely after fusion of chick myoblasts into myotubes [13]–[14]. Because we observed gradual redistribution of the majority of centrosome marker proteins onto the nuclear surface in differentiating myoblasts from mice [8], we studied the potential mechanisms of altering centrosome protein localization. In particular we wanted to address the following question: is there competition between the pericentriolar area and the nuclear surface for the binding of centrosome proteins during the differentiation process? If this is the case, then can re-introduction of new, fully assembled centrosomes into differentiated myotubes reverse microtubule organization and re-establish a centrosome-dependent microtubule network? Alternatively, are centrosomes gradually deactivated in the myotube, at later stages of the differentiation process?

**Figure 4:**
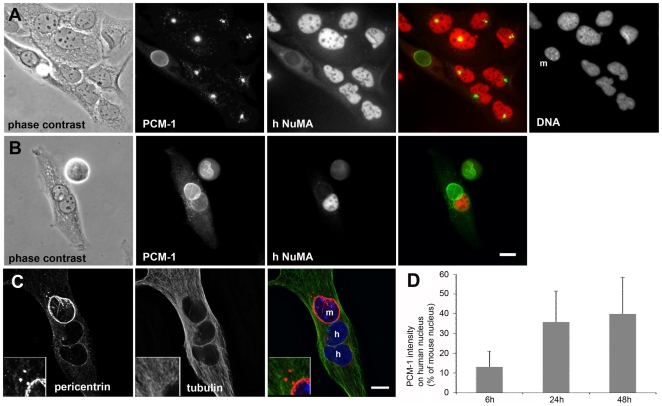
Centrosome proteins are recruited to the nuclear surface of non-muscle cells, after fusion with differentiated muscle cells. (A) Co-culture of *H-2K* ^b^-tsA58 mouse muscle cells with human U2OS cells. Top left: phase contrast; top middle: immunofluorescence of the centrosome protein PCM-1; top right: staining of the DNA with DAPI; bottom left: immunofluorescence of the human form of the nuclear protein NuMA; bottom right: merge of PCM-1 (green) and NuMA (red) staining. Note that NuMA staining is exclusively visible in nuclei of the human U2OS cells. Mouse nuclei (m) show characteristic condensation of heterochromatin in discrete nucleoplasmic punctuate staining that is absent from the surrounding human nuclei. (B) Heterokaryon, formed by fusion of a human U2OS cell with a mouse *H-2K* ^b^-tsA58 cell. A cell is shown at 48 hours after induction of fusion with polyethylene glycol. Staining and microscopy as in (A). The human nucleus, as identified by NuMA immunofluorescence, shows accumulation of perinuclear PCM-1. (C) Heterokaryon, 6 hours after induction of fusion, stained for pericentrin (red), tubulin (green), and DNA (blue). “m” and “h” indicate the nuclei contributed from the mouse *H-2K* ^b^-tsA58 cell, or from the human U2OS cells, respectively. Insets show an enlarged area of the cytoplasm, containing remnants of the centrosomes. (D) Histograms, showing PCM-1 intensity at human nuclei in heterokaryons, as a percentage of PCM-1 immunofluorescence levels around mouse nuclei from the same heterokaryon (6 h: n = 20; 24 h: n = 45; 48 h: n = 22). Bars in (B), (C), 10 µm.

To answer these questions, we designed an experiment in which we fused differentiated *H-2K* ^b^-tsA58 cells with non-muscle cells containing an active centrosome. We chose cells of the human osteosarcoma line U2OS, a cell type with distinct radial microtubule organization from the centrosome, indicating a pronounced centrosomal activity [15]. Cell fusion was induced with polyethylene glycol. In fused cells we distinguished human nuclei from mouse *H-2K* ^b^-tsA58 nuclei by the selective reactivity of a monoclonal antibody against the human form of the nuclear protein NuMA. In cultures of unfused *H-2K* ^b^-tsA58 and U2OS cells, the antibody stained specifically the nuclei of the human U2OS cells, but did not cross-react with the nuclei of mouse *H-2K* ^b^-tsA58 cells (Fig. 4A). The mouse nuclei were also recognizable by their distinct pattern of heterochromatin, which is organized in several dense spots (Fig. 4A). The cells were further stained for immunofluorescence of the centrosome proteins PCM-1 and pericentrin.

**Figure 5:**
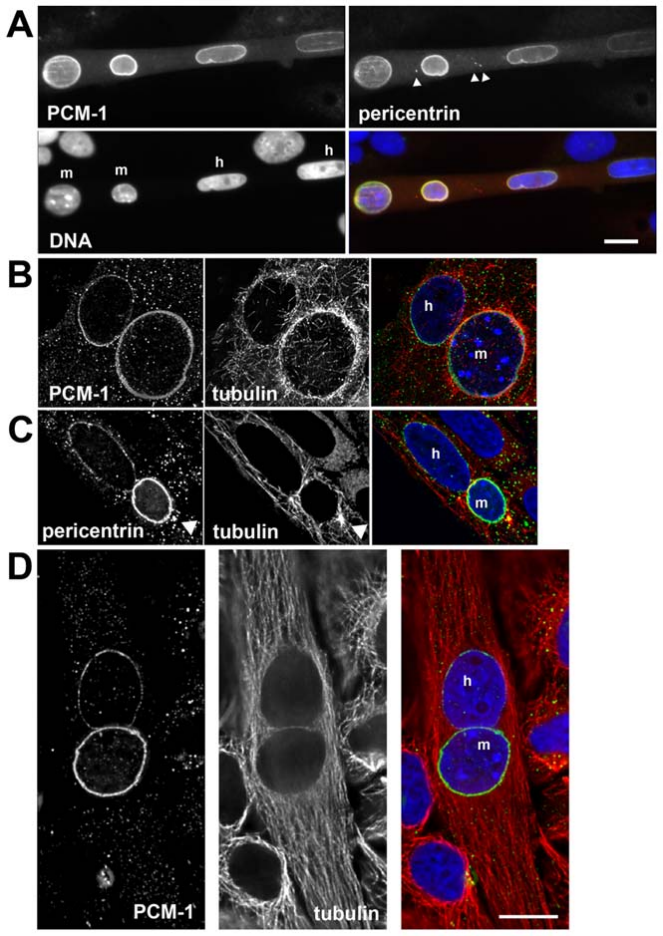
Microtubule nucleation from non-myoblast nuclei after fusion into heterokaryons with muscle cells. (A) Immunofluorescence of a heterokaryon of fused U2OS and *H-2K* ^b^-tsA58 cells, containing two mouse nuclei (m) and two human nuclei (h), as identified by chromatin morphology. The centrosome proteins PCM-1 and pericentrin are stained with antibodies, displayed in the merged image in green and red, respectively. Centrosomal foci of pericentrin are visible (arrowheads), in addition to perinuclear pericentrin staining. DNA is marked with DAPI (blue). (B, C) Microtubule re-growth is shown in heterokaryons at 48 hours after fusion, containing one human (h) and one mouse nucleus (m). Microtubules were re-grown for 20 seconds after cold-induced depolymerization. Immunofluorescence of PCM-1 (green), and tubulin (red) is shown. DNA (blue) is stained with DAPI. The arrowhead in (C) indicates the position of a centrosomal pericentrin focus, nucleating microtubules. (D) Immunofluorescence of a heterokaryon after full microtubule polymerization, stained as indicated in (B, C). Bars in (A), (D), 10 µm.

In differentiated cells of the *H-2K* ^b^-tsA58 cell line, PCM-1 and pericentrin accumulated around the nucleus, whereas in U2OS cells they marked the pericentriolar area prior to fusion (Fig. 4A). However, after fusion into heterokaryons, we observed that the nuclei of human origin also started to acquire PCM-1 and pericentrin at the nuclear surface (Fig. 4B, C). Early after fusion (six hours), only small amounts of PCM-1 or pericentrin accumulated around the human nuclei (Fig. 4C, D), whereas a more continuous perinuclear layer of these centrosome proteins was visible in many cells after 24 to 48 hours (Fig. 5A). Photometric analysis revealed that at 6 hours, human nuclei had acquired 13±8% of perinuclear pericentrin, as compared to mouse nuclei in the same heterokaryon. This amount increased to 36±16% after 24 hours, and remained at this level at 48 hours post fusion (40±19%; Fig. 4D). This suggests that perinuclear recruitment of centrosome proteins occurs rapidly within 24 hours, but reaches a plateau afterwards, and that recruitment is less efficient around the foreign nuclei than around the endogenous ones.

We performed microtubule re-growth experiments after cold-treatment, as described above, to test whether human nuclei that were surrounded by centrosome proteins were able to support microtubule nucleation. We found that re-polymerizing microtubules were indeed seen around these nuclei (Fig. 5B). In some heterokaryons, large pericentrin foci were seen in the cytoplasm, resembling centrosomes (Fig. 4C, Fig. 5A, B, arrowheads). Microtubule nucleation also occurred from these foci, as already described for early *H-2K* ^b^-tsA58 myotubes (Fig. 3A, Fig. 5C, arrowheads). After full polymerization of microtubules, non-radial orientation of microtubules was seen, with a preferential alignment along the long axis of the cytoplasm of the heterokaryon, as in regular myotubes (Fig. 5D). Although pericentrin foci were present in heterokaryons (Fig. 4C, Fig. 5A, C, arrowheads), no dominant centrosomal organization centres were detected (Fig. 4C, Fig. 5D). Moreover, the centrosome protein PCM-1 was absent from these foci (Fig. 5A).

### Perinuclear Accumulation of Centrosome Proteins Occurs Independently of Translation

**Figure 6:**
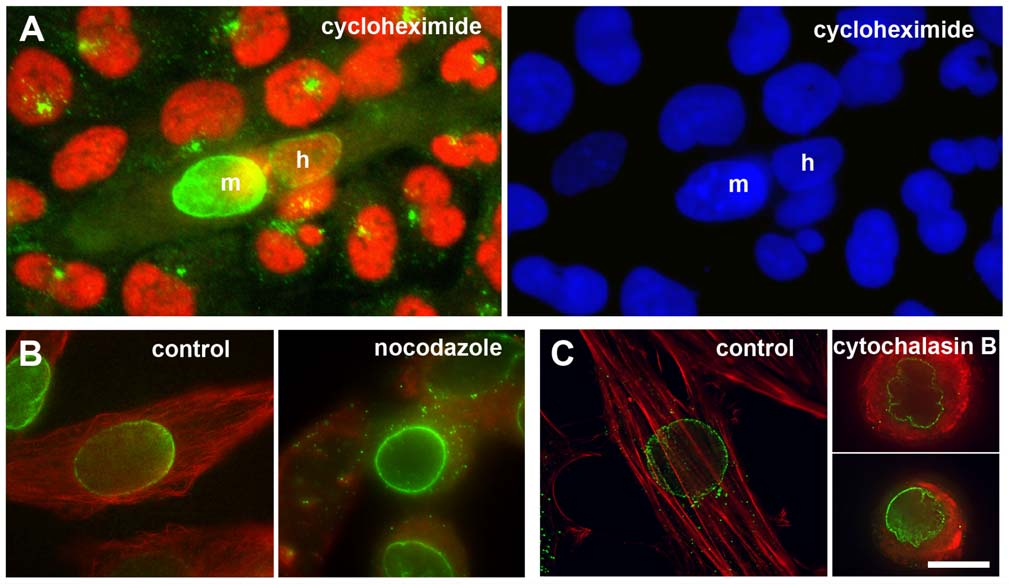
Recruitment of centrosome proteins to the perinuclear area occurs independently of new protein translation, or polymerized microtubules, or F-actin. (A) Heterokaryon of a fused U2OS cell with a differentiated *H-2K* ^b^-tsA58 cell, in the presence of 10 µM cycloheximide. Immunofluorescence of PCM-1 (green) and human NuMA (red) is shown. “m” and “h” indicate the nuclei contributed from the mouse *H-2K* ^b^-tsA58 cell, or from the human U2OS cell, respectively. A significant amount of the centrosome protein PCM-1 is seen accumulating around the human nucleus in the heterokaryon, but not in the unfused human U2OS cells. DNA is stained with DAPI (blue). (B) Left: differentiating control *H-2K* ^b^-tsA58 cell, stained for microtubules (red), and PCM-1 (green). Right: *H-2K* ^b^-tsA58 cell that differentiated for two days in the presence of 1 µM nocodazole, to depolymerize microtubules. (C) Left: differentiating control *H-2K* ^b^-tsA58 cell, stained with rhodamine-labelled phalloidin for polymerized actin (red), and with an antibody against PCM-1 (green). Right: two *H-2K* ^b^-tsA58 cells that differentiated for two days in the presence of 10 µg/ml cytochalasin B, to depolymerize actin. Bar in (c), 10 µm.

To examine whether the newly accumulated centrosome proteins around the human U2OS nuclei represented newly translated material or whether these proteins were recruited from the cytoplasmic pool of the heterokaryon, we repeated fusion experiments in the presence of the translation inhibitor cycloheximide (Fig. 6A). We discovered that even under these conditions, the centrosome protein PCM-1 accumulated at the perinuclear surface of U2OS nuclei upon fusion with differentiated *H-2K* ^b^-tsA58 cells, although slightly less efficiently: we found centrosome protein relocalization to the human nuclei in 55±9% (n = 33) of the heterokaryons treated with cycloheximide, in comparison to 71±6% (n = 63) of non-treated heterologous fusions.

In a first step towards identifying putative mechanisms of centrosome protein relocalization, we investigated the role of cytoskeletal elements such as microtubules and actin, to see whether perinuclear accumulation of centrosome proteins required cytoskeleton-dependent transport. As shown in Fig. 6(B, C), neither the depolymerization of microtubules by nocodazole, nor the depolymerization of actin with cytochalasin B or D prevented perinuclear assembly of PCM-1 in differentiating *H-2K* ^b^-tsA58 cells, suggesting that this assembly is independent of the cytoskeleton.

## Discussion

In this study, we provide insights into the mechanisms of centrosome reorganization in differentiating muscle cells. Large amounts of centrosome proteins relocalize to the nuclear surface at the onset of differentiation [6]–[8]. While the centrosome remains visible during the first few days of differentiation, with marker proteins such as pericentrin remaining partly at the pericentriolar material, the relative amount of microtubules nucleated from the centrosome diminishes. In contrast, in differentiating cells approximately 60% of microtubules are nucleated from the surface of the nucleus. This percentage remains stable during fusion of differentiating myoblasts into myotubes, whereas the percentage of centrosomal microtubules diminishes further. The loss of centrosomal nucleation may be due to loss of proteins from the pericentriolar material that are responsible for microtubule nucleation, and relocation of these proteins to the nuclear surface. This transfer may occur by dynamic exchange with a cytoplasmic pool of centrosomal proteins, as described in non-muscle cells [16]–[18]. Future work will be necessary to determine what nucleation factors, in addition to gamma-tubulin, are relocated to the nucleus [7].

Consistently, we find that salt-treated centrosomes, lacking microtubule nucleating factors, can be reconstituted by incubation with cytoplasmic extracts from myotubes. Thus, all components necessary for centrosomal nucleation are still present in the cytoplasm of differentiated cells. In addition to nuclear and centrosomal sites of microtubule nucleation, approximately one third of the microtubules in the cell seem to emanate from cytoplasmic sites. This is consistent with a previous report [10], describing microtubule nucleation in myotubes from multiple cytoplasmic sites.

During the process of differentiation, microtubule organization in differentiating myoblasts changes from a radial into a longitudinal, non-centrosomal orientation. In part, this may be directly due to reduced centrosomal nucleation. In addition, this may also be explained by loss of microtubule anchoring to the centrosome. This hypothesis is supported by the observation that centrosomal foci in myotubes and heterokaryons show accumulation of the protein pericentrin, but not PCM-1 ([8], Fig. 5A). Since it has previously been shown that in the absence of PCM-1 centrosomal anchoring of microtubules is prevented [15], it is possible that centrosomes in differentiating myoblasts lose a specific subset of proteins from their pericentriolar material, thus impeding them from anchoring microtubules, besides reducing their capacity of nucleation.

Moreover, our results on heterologous cell fusion between muscular and non-muscular cells demonstrate that the centrosomes of the undifferentiated non-muscle cells do not play a dominant role as microtubule organizing centres in the heterokaryons. Rather, our data suggest that nuclei of non-muscular origin acquire the potential to bind centrosome proteins, once exposed to a differentiated, muscular environment. Our experiments with the inhibitor cycloheximide indicate that in fused cells, centrosome proteins are recruited to the nuclear envelope independent of any additional transcriptional/translational event: (i) either by transfer from centrosomes or other nuclei that have already enriched these proteins, or (ii) from a cytoplasmic pool. Several centrosome proteins in non-muscle cells, including centrin and gamma-tubulin, are known to repartition between both a soluble, cytoplasmic pool, and an insoluble, centrosome-bound pool [16]–[17]. The soluble pool contains 80% or more of the respective protein, and soluble protein is in dynamic exchange with centrosome-bound protein [18]. Likewise, in a differentiating muscular cell, the nuclear surface may recruit centrosome proteins from the large cytoplasmic pool. Moreover, the nuclear surface may represent a default site of centrosome protein accumulation and microtubule nucleation, such as seen in other biological systems such as plant cells [19].

**Figure 7:**
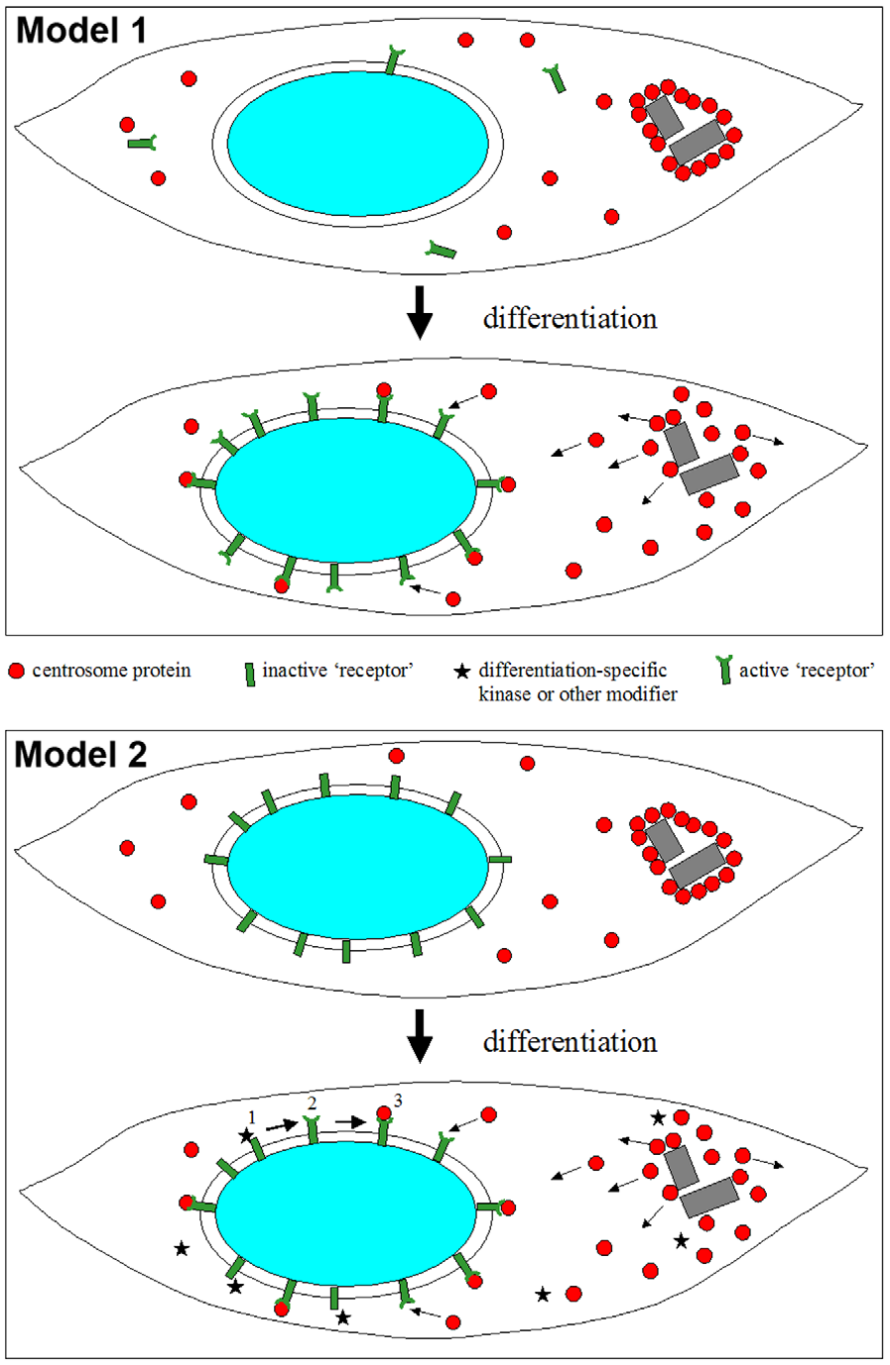
Potential mechanisms of centrosome protein relocalization in differentiating muscle cells. Model 1: during differentiation of myoblasts, ‘receptor’ proteins (green) are expressed that bind to the nuclear surface and that recruit centrosome proteins (red). Centrosome proteins that are found enriched at the pericentriolar material prior to differentiation are transferred to the nuclear surface via dynamic exchange through a soluble cytoplasmic pool. Because the sequestration of centrosome proteins to the nuclear surface lowers the concentration of the soluble cytoplasmic pool, and because this pool is in an equilibrium with centrosome proteins at the pericentriolar material, sequestration to the nucleus will indirectly lead to the disassembly of the pericentriolar material (small arrows), and finally to the loss of centrosomal activity. Model 2: in undifferentiated myoblasts (top), nuclei possess putative ‘receptor’ proteins (green) at their outer surface that become activated only at the onset of differentiation (bottom). (1) This activation may be mediated by specific kinases, or by other factors involved in posttranslational modification (stars). (2) As a consequence, the receptors are now competent to bind centrosome proteins to the nuclear surface (3).

In addition, our experiments indicate that any factors triggering nuclear recruitment of centrosome proteins must have been restricted to the differentiated *H-2K* ^b^-tsA58 cell prior to fusion, since no perinuclear accumulation of centrosome proteins was seen in unfused human U2OS cells or in undifferentiated myoblasts. We therefore propose two possible mechanisms: (1) upon differentiation, muscle cells express ‘receptors’ that are anchored to the nuclear surface and that bind centrosome proteins (Fig. 7A). In our fusion experiments, these ‘receptors’ may be present in abundance in myotubes, but absent from non-muscle cells. Fusion would permit these receptors to associate with the nuclear envelope of the non-muscle nucleus and to recruit centrosome proteins. (2) A second possibility would be that undifferentiated cells, independent of their origin, possess putative ‘receptors’ for centrosome proteins at the outer surface of the nucleus, and that in a differentiating environment of a muscle cell, the recruitment of centrosome proteins would be stimulated by activating these receptors with the help of differentiation-specific factors, such as kinases or other proteins involved in posttranslational modification (Fig. 7B). Moreover, specific transport or docking proteins may be involved in the relocalization of centrosome proteins. In future experiments, it will be important to identify putative factors and nuclear receptors involved in centrosome protein relocation, since the reorganization of microtubules and microtubule-organizing proteins has been shown to be an important event in myoblast differentiation, elongation and fusion [12], [20].

## Materials and Methods

### Cell Culture and Fusion Experiments

U2OS cells were grown in Dulbecco's modified Eagle's medium, containing 10% fetal calf serum. C2C12 cells were grown as described [8]. Mouse *H-2K* ^b^-tsA58 myoblasts carrying a thermolabile T-antigen were grown at 33°C in Dulbecco's modified Eagle's medium, 2% chicken embryonic extract, 20% fetal calf serum and 20 I.U. interferon-gamma [10]. Differentiation of *H-2K* ^b^-tsA58 cells was induced by temperature shift to 37°C and simultaneous serum starvation, by replacing the regular growth medium with Dulbecco's modified Eagle's medium containing only 2% fetal calf serum. *H-2K* ^b^-tsA58 cells were differentiated for five days, to obtain a high yield of differentiated myotubes. After removal of the culture medium, cells were treated with 1x Trypsin/EDTA (Gibco) for several seconds, leading to selective detachment of myotubes. Detachment was monitored by phase contrast microscopy. Detached cells were collected and trypsin was neutralized by addition of growth medium. Cells were then seeded with exponentially growing U2OS cells. The following day, cells were washed three times with pre-warmed phosphate-buffered saline (PBS), then incubated one minute with pre-warmed polyethylene glycol 1500 (Boehringer Mannheim) to induce cell fusion. Polyethylene glycol was removed by washing extensively with PBS, and the cells were then grown in Dulbecco's modified Eagle's medium, containing 5% fetal calf serum.

### Immunofluorescence Staining

Cells were grown on glass coverslips and fixed in methanol at –20°C for 10 minutes. Immunofluorescence was performed using standard procedures. Antibodies used in this study were from rabbit and mouse against PCM-1 [15], rabbit anti-EB3 [12], mouse monoclonal anti-pericentrin [15], rabbit polyclonal anti-pericentrin (Covance), monoclonal antibody GTU-88 against gamma-tubulin (Sigma), monoclonal antibody DM1a against alpha-tubulin (Sigma), and monoclonal antibody Ab-2 against NuMA (Calbiochem). DNA was stained with 4′,6-diamidino-2-phenylindole (DAPI).

Quantification of microtubule nucleation was performed in cells recovering from cold treatment (3 to 4 hours on ice), at 10 seconds after shifting the temperature to 37°C. For this, cells were fixed and stained for immunofluorescence of EB3 and pericentrin. Three-dimensional data sets of entire cells were acquired with a Deltavision RT microscope (Applied Precision), using Olympus 100x/NA 1.40, 60x/NA 1.42, and 40x/NA 1.30 lenses. Optical stacks were recorded at 0.2 µm intervals, and deconvolved using Softworx 3.5.0 software (Applied Precision). Comets of EB3 were tracked and counted on a sheet of transparent plastic attached to the computer screen, using coloured marker pens to record nuclear, centrosomal, and free cytoplasmic EB3, while scrolling through the stack. Pericentrin immunofluorescence was quantified within a rim of 1 µm at the nuclear envelope, using the ‘select’ tool and the histogram function of Adobe Photoshop.

### Nucleation of Microtubules In Vitro

Centrosomes were purified from Jurkat cells as described previously [21]. Purified centrosomes were incubated in 1 M potassium iodide (KI) at 4°C in the dark for 15, 30, 45, and 60 minutes, then centrifuged at 120,000 *g* for 30 minutes at 4°C. The KI-soluble material was then concentrated and filtered using a Centricon YM-10 (Millipore) device. The retained proteins were recovered, boiled for 5 minutes in protein sample buffer and stored at –80°C until loading onto 7.5% Tris-glycine polyacrylamide gels. For the preparation of cytoplasmic extracts from muscle cells, *H-2K* ^b^-tsA58 cells or C2C12 cells were used. The degree of differentiation was assessed by immunofluorescence of the marker embryonic myosin (data not shown). Undifferentiated cultures and cultures after 5 days of induction, containing at least 81% of differentiated cells, were processed. To prepare cytoplasmic extracts, *H-2K* ^b^-tsA58 cells or C2C12 cells were washed twice in cold PBS. Subsequently, the cells were washed in 50 ml of cold KPN buffer (50 mM KCl, 50 mM PIPES pH 7.0, 10 mM EGTA, 1.92 mM MgCl_2_, 1 mM DTT, 100 µM PMSF, 20 µM cytochalasin B, 10 µg/ml of leupeptin, pepstatin, chymostatin), then in 1 ml of KPN buffer. After centrifugation at 800 g, the pellet of cells was frozen in liquid nitrogen. Cells were lysed by three cycles of thawing-freezing, and ground using a pellet pestle. The lysate was then separated by ultra-centrifugation at 120,000 g for 45 minutes at 4°C, and the soluble supernatant was collected. Centrosomes were spun onto glass coverslips of 12 mm diameter as described [22]. Coverslips were incubated on ice for one hour with 20 µl of cytoplasmic supernatant from myoblasts, myotubes, or with buffer alone. After removal of the extract or buffer, coverslips were incubated for 10 minutes with pure porcine brain tubulin at 5 mg/ml (Cytoskeleton Inc.), supplemented with rhodamine-labelled tubulin (Cytoskeleton Inc.). Microtubules were fixed as described [22], and viewed under a fluorescence microscope.
